# Phase angle is related to physical function and quality of life in preoperative patients with lumbar spinal stenosis

**DOI:** 10.1038/s41598-023-40629-0

**Published:** 2023-08-25

**Authors:** Ryota Otsubo, Ryuki Hashida, Kenta Murotani, Sohei Iwanaga, Keisuke Hirota, Shunji Koya, Yuya Tsukada, Yuta Ogata, Kimiaki Yokosuka, Tatsuhiro Yoshida, Ichiro Nakae, Takuma Fudo, Shinji Morito, Takahiro Shimazaki, Kei Yamada, Kimiaki Sato, Hiroo Matsuse, Naoto Shiba, Koji Hiraoka

**Affiliations:** 1 Division of Rehabilitation, Kurume University Hospital, 67 Asahi-machi, Kurume, Fukuoka Japan 830-0011; 2 Department of Orthopaedics, Kurume University School of Medicine, 67 Asahi-machi, Kurume, Fukuoka Japan; 3 Biostatistics Center, Kurume University, 67 Asahi-machi, Kurume, Fukuoka Japan; 4 Institute of Health and Sports Sciences, Kurume University, Kurume, Japan; 5 Division of Rehabilitation, Kurume University Medical Center, Kurume, Japan

**Keywords:** Biomarkers, Health care, Medical research

## Abstract

Lumbar spinal stenosis (LSS) can interfere with daily life and quality of life (QOL). Evaluating physical function and QOL and helping patients to improve is the focus of rehabilitation. Phase angle (PhA) assessment is widely used to measure body composition and is considered an indicator of physical function and QOL. This study investigated the relationship between PhA and physical function, physical activity, and QOL in patients with LSS. PhA, handgrip strength, walking speed, Timed Up and Go test (TUG), Life Space Assessment (LSA), Prognostic Nutritional Index (PNI), Japanese Orthopaedic Association Back Pain Evaluation Questionnaire (JOABPEQ), and EQ-5D were assessed and statistically analyzed. The study included 133 patients with LSS. Multiple regression analysis of PhA adjusted for age, sex, and body mass index (Model 1) and for Model 1 + PNI (Model 2) showed significant correlations (P < 0.05) with handgrip strength, walking speed, TUG, and LSA. Regarding QOL, PhA was significantly correlated (P < 0.05) with lumbar function in JOABPEQ. PhA was associated with physical function and QOL in patients with LSS and might be a new clinical indicator in this population.

## Introduction

Lumbar spinal stenosis (LSS) is caused by the narrowing of the spinal canal, impeding the nerves and blood vessels traveling through the lumbar spine. LSS causes pain in the lower back, buttocks, and lower extremities, which can interfere with daily life^1^. Intermittent claudication is one of the most common symptoms of LSS, severely limiting the patient’s mobility. LSS impairs activities of daily living and may lead to psychosocial problems resulting from depression and isolation due to symptoms such as intermittent claudication, back pain, numbness, and muscle weakness in the lower limbs, leading to a decreased quality of life (QOL)^2^. Improving QOL is one of the important goals of LSS treatment^3^, and assessing QOL is important in physical therapy.

In recent years, phase angle (PhA) assessment has been widely used to noninvasively measure body composition and has been reported as a measure of skeletal muscle quality^4^. A high PhA reflects higher cellularity, cell membrane integrity, and better cell function, whereas a low PhA reflects cell loss and reduced cell membrane integrity^5^. PhA has been reported to be associated with physical functions such as muscle strength, balance, and walking speed in both community-dwelling elderly individuals and cancer patients^6,7^. An association between PhA and health-related QOL has been reported in patients on maintenance hemodialysis^8,9^. Therefore, PhA is an indicator of physical function and QOL and is considered a useful tool for assessing the patient’s condition in clinical practice. However, although PhA is a noninvasive and simple method of measuring body composition, few studies have used PhA in orthopedic diseases, and its relationship with physical function and QOL has not been fully investigated. LSS is often associated with pain and numbness, which may make it difficult to assess physical functions such as muscle strength, balance function, and walking speed. In addition, physical function, activities of daily living, and QOL are interrelated, and PhA, which can be measured noninvasively, may be useful for the indirect assessment of physical function and QOL in patients who are difficult to assess in practice.

Therefore, this study aimed to investigate the relationship between PhA, physical function, and QOL in patients with LSS.

## Methods

### Ethics

The study protocol conformed to the ethics guidelines of the Declaration of Helsinki, as reflected in the prior approval given by the institutional review board of Kurume University Hospital (approval ID: 22017). This study was retrospective; thus, we provided informed consent forms including the study title, subject, and aim on the web. Informed consent from patients was obtained using an opt-out approach.

### Patients

This cross-sectional study included 191 patients (age ≥ 60 years) admitted for surgery for LSS in the orthopedic ward of our hospital between May 2020 and April 2021. Data collection was conducted in May 2022, and no personally identifiable participant information was accessed during the data collection. We excluded patients with pyogenic spondylitis, metastatic bone tumor, history of stroke, difficulty standing due to hemiplegia, difficulty measuring body composition, insertion of a pacemaker, and inability to answer the questionnaire. PhA was measured before admission, and physical function and QOL were assessed before surgery.

### Phase angle (PhA)

PhA was measured using bioelectrical impedance analysis (BIA). An Inbody 770 BIA unit (Inbody Co., Ltd., Seoul, Korea) was used for the measurements. BIA measures body composition by passing a weak alternating current through the body and taking the impedance or the difference between each tissue. Impedance is a general term for resistance components that interfere with the current, and impedance can be divided into resistance (R) and reactance (Xc). PhA is the value of arctangent, which is Xc divided by R, and is expressed by the following equation^5^:

$$Pha = arctangent \times Xc/R \times 180/\pi$$

### Life space assessment (LSA)

LSA is an index that assesses mobility in the spatial extent of an individual’s daily life^10^. This consists of the range of living space, frequency, presence of assistive devices, and level of independence, and it is a measure that investigates the range of activities in the living space over the past month. The living space from the bedroom at home to outside the town is divided into five levels, and the total score is calculated by multiplying the score of each level by the frequency and degree of independence. The maximum score is 120, with higher scores indicating a higher level of activity. LSA has been used to assess mobility and activities of daily living in a variety of populations, including community-dwelling elderly individuals and stroke patients^11,12^.

### Handgrip strength

Handgrip strength was measured using a Smedley-type grip strength meter (Takei Kiki Kogyo, T.K.K. 5101)^13^. The subject was instructed, “After the signal, grip the grip strength meter as strongly as possible. Please hold the grip strength meter for 3 s.” The right hand was measured twice, and the maximum value was adopted. Grip strength is used to measure voluntary muscle function as an indicator of muscle strength in patients with LSS^14^.

### Walking speed

A 10-m walk test was conducted to calculate the walking speed. In the 10-m walk test, two marks were placed on the floor so that the distance was 10 m in a straight line, and the time from the leading foot passing the first mark to the moment it stepped on or over the second mark was measured with a stopwatch. To ensure the subject started 1 m before the first mark and passed the second mark by 1 m, the subject was not told about the marks but was shown the direction and instructed, “Walk straight ahead at your normal walking speed^15^”.

### Timed Up and Go test (TUG)

For the TUG, the subject was seated and asked: “Please get up from the chair, walk as fast as you can, walk around the cone 3 m ahead, and sit on the chair again. You may turn in either direction.” The TUG time was measured with a stopwatch from the time the subject’s body began to move till the time they were seated on the chair again. A chair with a height of 42.0 cm and an elbow rest was used^16^.

### Prognostic nutritional index (PNI)

PNI is a simple and reliable assessment of nutritional status^17^. Consequently, PNI was assessed in this study because it is considered a confounder of nutrition. PNI is a prognostic score that is calculated by reflecting both the inflammatory and nutritional status of the patient. In the current study, it was calculated via the following formula using the preoperative blood test values.

$$\left\{10\times \; serum \; albumin\left(\text{g}/\text{L}\right)+\left(0.005\times total \; lymphocyte \;count(/\upmu \text{L})\right)\right\}$$

PNI has been reported to be a useful marker of malnutrition^18^. It has also been reported that a low preoperative PNI indicates a low nutritional status and is associated with postoperative complications in spinal diseases^19,20^.

### Japanese Orthopaedic Association Back Pain Evaluation Questionnaire (JOABPEQ)

JOABPEQ is a back-pain disease-specific QOL assessment that combines the Roland-Morris Disability Questionnaire (RDQ) and the Short-Form Health Survey (SF-36) with the Visual Analog Scale (VAS). The assessment consists of 25 items corresponding to five subscales: low back pain (4 items), lumbar function (6 items), walking ability (5 items), social life function (3 items), and mental health (7 items). The score for each subscale ranges from 0 to 100, with higher scores indicating better conditions^21^.

### EuroQOL 5 dimensions 3-level (EQ-5D)

The EQ-5D is a comprehensive QOL scale that can be calculated by answering five questions on three levels: “degree of mobility,” "personal care," "usual activities," "pain/discomfort," and "anxiety/stress.” It has been used to assess health-related QOL in middle-aged and elderly people living at home and for various diseases, including orthopedic diseases^22,23^.

### Statistical analysis

JMP version 15.0 was used for the statistical analysis, and the significance level was set at 5%. The association between PhA and each measurement item (age, sex, body mass index [BMI], PNI, handgrip strength, walking speed, TUG, LSA, each sub-item of JOABPEQ, and EQ-5D) was investigated using Spearman's rank correlation. PhA is influenced by three main factors: age, sex, and BMI^5^, and it is considered a useful marker of malnutrition^18^. In the present study, we focused on the relationship of PhA with physical function and QOL. For this purpose, we created two models, one without adjustment for PNI (Model 1) and the other with adjustment for PNI (Model 2). With each model, we investigated the relationship of PhA with physical function (grip strength, walking speed, TUG), physical activity (LSA), and QOL (JOABPEQ subitems, EQ-5D) using multiple regression analysis, with PhA as the dependent variable. Grip strength, TUG, and 10-m walk tests are easy and practical tools for assessing patients' physical abilities, and these physical assessments have been used in patients with LSS in clinical settings^14,24,25^. Thus, we applied them in this study.

## Results

### Patients

**Figure 1:**
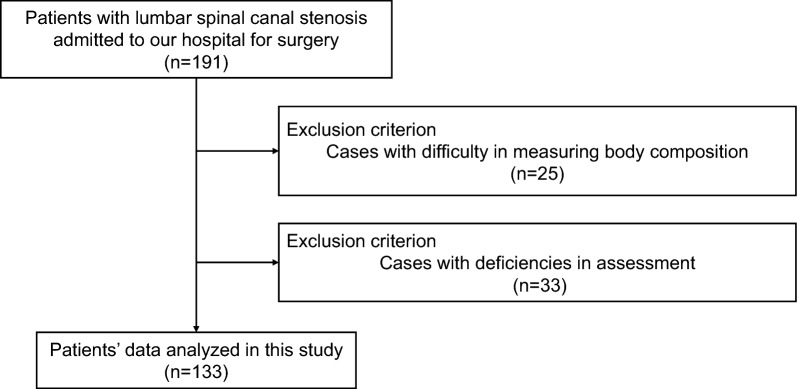
Flowchart of the patient inclusion process.

Of the 191 patients who met the eligibility criteria, 133 were analyzed after the exclusion criteria were applied (Fig. 1). The basic attributes of the patients are shown in Table 1.

**Table 1:** Patient characteristics.

Variable	
Total patients	133
Age	75 (87–65)
Sex (M/F)	61/72
BMI (kg/m^2^)	23.10 (15.37–32.14)
PhA	4.3 (3.1–6.0)
PNI	51.5 (35.9–64.9)
Handgrip strength	22.8 (8.1–45.7)
Walking speed	0.945 (0.59–2.22)
TUG	9.6 (4.9–46.6)
LSA	50 (1–120)
JOABPEQ (low back pain)	43 (0–100)
JOABPEQ (lumbar function)	58 (0–100)
JOABPEQ (walking ability)	29 (0–100)
JOABPEQ (social life function)	43 (3–100)
JOABPEQ (mental health)	45 (0–89)
EQ-5D	0.592 (0.280–1)
VAS (low back pain)	55 (0–95)
VAS (lower limb pain)	64 (0–95)
VAS (lower limb numbness)	58 (0–100)

Values are provided as number or median (min–max).

PhA, phase angle; BMI, body mass index; PNI, Prognostic Nutritional Index; TUG, Timed Up and Go test; LSA, Life Space Assessment; JOABPEQ, Japanese Orthopaedic Association Back Pain Evaluation Questionnaire; EQ-5D, EuroQOL 5 dimensions 3-level; VAS, Visual Analog Scale.

### Correlation between PhA and each measurement item

The results of Spearman's rank correlation analysis of the relationship between PhA and each measurement item are shown in Table 2. PhA was positively correlated with BMI, PNI, handgrip strength, walking speed, and LSA and negatively correlated with age, TUG, and VAS (lower limb pain).

**Table 2:** Spearman's rank correlation analysis (PhA vs. measurement items).

Variable	vs Variable	ρ	P-value
Phase angle	Age	− 0.387	< 0.0001*
	BMI	0.177	0.0419*
	PNI	0.197	0.0241*
	Handgrip strength	0.575	< 0.0001*
	Walking speed	0.455	< 0.0001*
	TUG	− 0.470	< 0.0001*
	LSA	0.479	< 0.0001*
	JOABPEQ (low back pain)	− 0.003	0.9730
	JOABPEQ (lumbar function)	0.258	0.0027*
	JOABPEQ (walking ability)	0.195	0.0243*
	JOABPEQ (social life function)	0.213	0.0136*
	JOABPEQ (mental health)	0.270	0.0016*
	EQ-5D	0.206	0.0197*
	VAS (low back pain)	− 0.030	0.7371
	VAS (lower limb pain)	− 0.168	0.0567^†^
	VAS (lower limb numbness)	0.054	0.5420

PhA, phase angle; BMI, body mass index; PNI, Prognostic Nutritional Index; TUG, Timed Up and Go test; LSA, Life Space Assessment; JOABPEQ, Japanese Orthopaedic Association Back Pain Evaluation Questionnaire; EQ-5D, EuroQOL 5 dimensions 3-level; VAS, Visual Analog Scale.

P-value < 0.1^†^, P-value < 0.05*.

Regarding QOL assessment, PhA was positively correlated with lumbar function, walking ability, social life function, and mental health, which are JOABPEQ subitems, and with EQ-5D.

The results of the Wilcoxon signed-rank test of the relationship between PhA and each measurement item are shown in Table 3. The patients were divided into two groups using a median of PhA. The male/female ratio, hand grip strength, walking speed, TUG, LSA, JOABPPEQ (lower limb pain, mental health), and EQ-5D were significantly higher in the high PhA group than in the low PhA group. Age and VAS score (lower limb pain) were significantly lower in the high PhA group than in the low PhA group.

**Table 3:** Wilcoxon signed-rank test (PhA vs. measurement items).

	High PhA (N = 72)		Low PhA (N = 61)		
	Median	Range (min–max)	Median	Range (min–max)	P-value
Age	74	65–86	77	65–87	0.0011*
Sex (M/F)	45/27		16/45		< 0.0001*
BMI (kg/m^2^)	23.35	17.58–32.14	23.01	15.37–31.22	0.1198
PNI	51.95	36.9–64.9	50.5	35.9–60.5	0.1971
Handgrip strength	29.8	11.3–45.7	19	8.1–35.3	< 0.0001*
Walking speed	1.055	0.43–2.22	0.825	0.39–2.06	< 0.0001*
TUG	8.84	4.9–36.4	11.6	6.38–46.6	< 0.0001*
LSA	62	4–120	40	1–120	< 0.0001*
JOABPEQ (low back pain)	43	0–100	43	0–100	0.7886
JOABPEQ (lumbar function)	58	8–100	50	0–100	0.0470*
JOABPEQ (walking ability)	29	0–100	21	0–71	0.0822^†^
JOABPEQ (social life function)	46	3–100	38	3–73	0.1208
JOABPEQ (mental health)	48	6–89	42	0–72	0.0053*
EQ-5D	0.596	0.37–1	0.58	0.28–1	0.0036*
VAS (low back pain)	52.5	0–95	56.5	0–95	0.8426
VAS (lower limb pain)	52.5	0–95	56.5	0–95	0.0107*
VAS (lower limb numbness)	58	0–100	58	0–95	0.8965

PhA, phase angle; M, Male; F, Female; BMI, body mass index; PNI, Prognostic Nutritional Index; TUG, Timed Up and Go test; LSA, Life Space Assessment; JOABPEQ, Japanese Orthopaedic Association Back Pain Evaluation Questionnaire; EQ-5D, EuroQOL 5 dimensions 3-level; VAS, Visual Analog Scale.

P-value < 0.1^†^, P-value < 0.05*.

### Multiple regression analysis of confounding-adjusted PhA, physical function, and QOL

Table 4 shows the results of the multiple regression analysis of the relationship of PhA with physical function and physical activity (adjusted for age, sex, and BMI [Model 1] and age, sex, BMI, and PNI [Model 2]). Table 5 shows the results of multiple regression analysis of the relationship between PhA and QOL (Models 1 and 2).

**Table 4:** Multiple regression analysis (PhA vs physical activity and physical function).

	Outcome	Model	Estimated value	95%CI	P-value	standardized β	VIF
Physical activity	LSA	1	0.007	0.003–0.010	< 0.0001*	0.317	1.203
		2	0.002	0.004–0.011	< 0.0001*	0.345	1.221
Physical function	Handgrip strength	1	0.035	0.022–0.049	< 0.0001*	0.522	2.382
		2	0.034	0.020–0.048	< 0.0001*	0.509	2.439
	Walking speed	1	0.529	0.304–0.755	< 0.0001*	0.312	1.073
		2	0.559	0.340–0.779	< 0.0001*	0.334	1.081
	TUG	1	− 0.024	− 0.036 to − 0.012	0.0002*	− 0.269	1.093
		2	− 0.022	− 0.034 to − 0.010	0.0003*	− 0.258	1.102

PhA, phase angle; CI, confidence interval; VIF, Variance Inflation Factor; LSA, Life Space Assessment; TUG, Timed Up and Go test; BMI, body mass index; PNI, Prognostic Nutritional Index.

Model 1: Adjusted for age, sex, and BMI.

Model 2: Adjusted for age, sex, BMI, and PNI.

P-value < 0.05*.

**Table 5:** Multiple regression analysis (PhA vs QOL).

	Outcome	Model	Estimated value	95%CI	P-value	standardized β	VIF
Disease-specific QOL	JOABPEQ (low back pain)	1	− 0.000	− 0.003 to 0002	0.7721	− 0.021	1.004
		2	− 0.000	− 0.003 to 0.002	0.8184	− 0.016	1.011
	JOABPEQ (lumbar function)	1	0.004	0.001 to 0.007	0.0078*	0.196	1.111
		2	0.004	0.001 to 0.007	0.0090*	0.194	1.112
	JOABPEQ (walking ability)	1	0.004	0.001 to 0.008	0.0153*	0.171	1.016
		2	0.005	0.002 to 0.009	0.0055*	0.197	1.027
	JOABPEQ (social life function)	1	0.003	− 0.001 to 0.008	0.1740	0.100	1.097
		2	0.004	− 0.001 to 0.009	0.0787^†^	0.132	1.122
	JOABPEQ (mental health)	1	0.006	0.001 to 0.011	0.0193*	0.166	1.021
		2	0.007	0.001–0.012	0.0135*	0.176	1.027
Comprehensive QOL	EQ-5D	1	0.357	− 0.392 to 1.105	0.3474	0.068	1.064
		2	0.458	− 0.297 to 1.212	0.2319	0.088	1.076

PhA, phase angle; QOL, quality of life; CI, confidence interval; VIF, Variance Inflation Factor; JOABPEQ, Japanese Orthopaedic Association Back Pain Evaluation Questionnaire; EQ-5D, EuroQOL 5 dimensions 3-level; BMI, body mass index; PNI, Prognostic Nutritional Index.

Model 1: Adjusted for age, sex, and BMI.

Model 2: Adjusted for age, sex, BMI, and PNI.

P-value < 0.1^†^, P-value < 0.05*.

Regarding physical function and physical activity, significant correlations were found between PhA and handgrip strength, walking speed, TUG, and LSA, and for QOL, significant correlations were found between PhA and the lumbar function, walking ability, and mental health subitems of JOABPEQ in both Model 1 and 2. However, no correlation with EQ-5D was observed.

## Discussion

**Figure 2:**
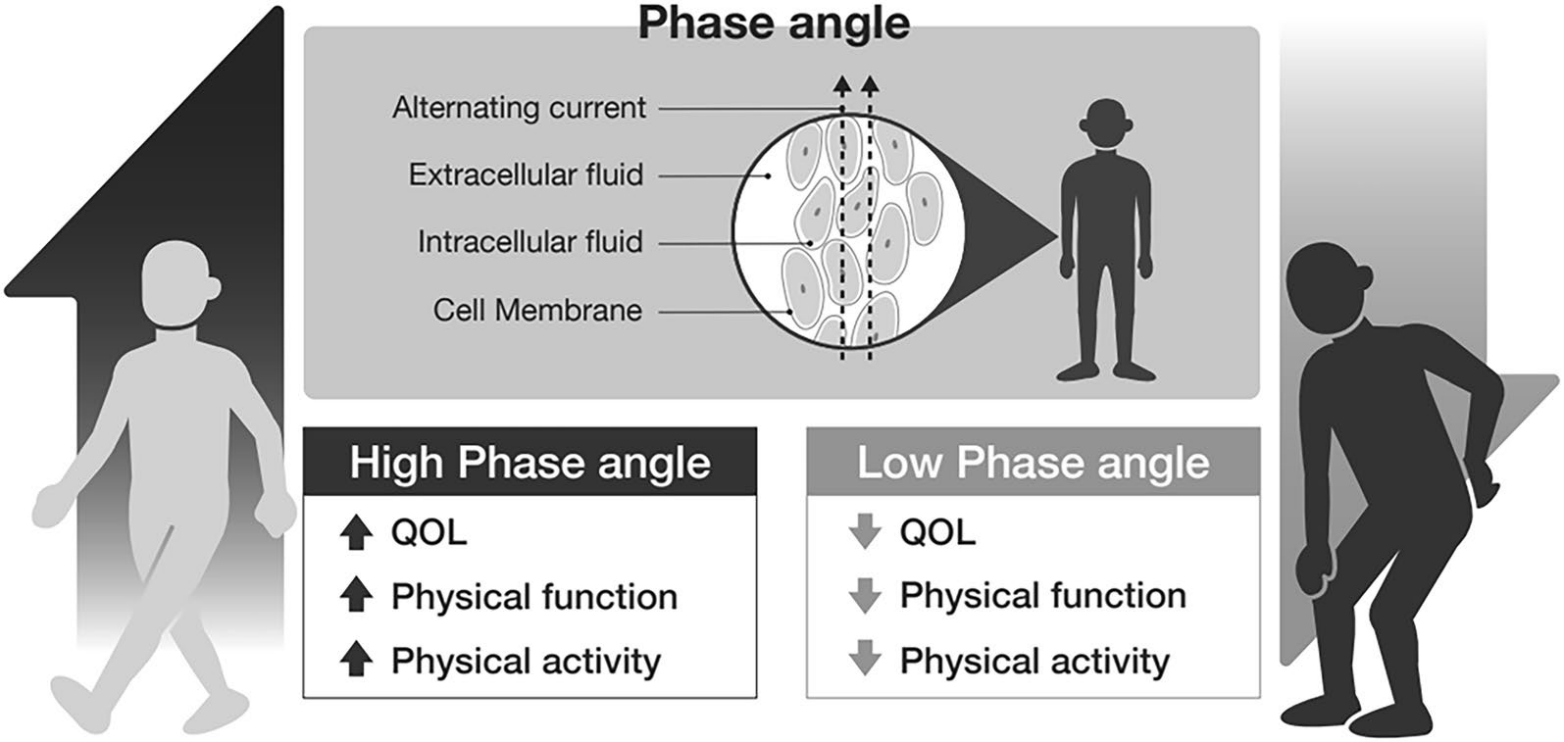
Summary of the study. The phase angle using bioelectrical impedance analysis was associated with quality of life, physical function, and physical activity in patients with lumbar spinal stenosis.

This study investigated the relationship between PhA and physical function, physical activity, and QOL in LSS patients admitted for surgery. A significant correlation was found between PhA and physical function and physical activity, and PhA was correlated with the subitems of JOABPEQ, a disease-specific QOL assessment, and EQ-5D, a comprehensive QOL assessment. Furthermore, in the multiple regression analysis, both Model 1 and Model 2 showed that PhA was correlated with the lumbar function subitem of JOABPEQ. PhA may be a useful tool for clinical and perioperative evaluation because it reflects both physical and QOL (Fig. 2).

### Relationship between PhA, physical function, and physical activity

In the present study, significant positive correlations were found between the PhA of LSS patients and handgrip strength and walking speed, and significant negative correlations were found with the TUG test. Yamada et al. reported that PhA was significantly correlated with lower limb muscle strength in community-dwelling elderly persons^26^. Tanaka et al., in their study of the locomotive syndrome (LS) and PhA in community-dwelling elderly persons, found that PhA was significantly correlated with the TUG test and was an independent factor for LS^27^. In the present study on LSS and in previous studies on other diseases, PhA was significantly higher in patients with higher physical function.

Furthermore, in the present study, a significant positive correlation was found between PhA and LSA, which is an indicator of physical activity. High physical activity is associated with high physical function, and a correlation between physical activity and PhA has been reported in other studies^17,28^. However, the mechanisms underlying the relationship between physical activity, physical function, and PhA are unknown. Considering the principles of PhA measurement, it is likely that the results of the present study were affected by the decrease in the amount of fat. Fat has high electrical resistance, and physical activity decreases this resistance. Moreover, the increase in the size and mass of cells and the structural integrity of cell membranes increases the reactance (Xc), resulting in a higher PhA^5^.

### PhA and JOABPEQ

PhA was correlated with lumbar function in JOABPEQ in both Model 1 and 2. The JOABPEQ items for lumbar function included the following: "I sometimes ask for help when I do something because of my back pain," "I try not to bend my back or kneel because of my back pain," "I have difficulty getting up from a chair because of my back pain," "I have difficulty turning over because of my back pain," "I have difficulty putting on socks or stockings because of my back pain” and "Do you find it difficult to bend forward, kneel, or bend over because of your physical condition?” These questions are related to the flexion, extension, and rotation of the trunk. For preoperative LSS patients with low back pain, trunk flexion, extension, and rotation movements should not be assessed because they are likely to induce pain. Therefore, PhA may be one of the indicators for evaluating lumbar function in patients with LSS because it can be used to estimate lumbar function without actual movements.

### PhA and quality of life

There was a significant association between PhA and the lumbar function subitems of JOABPEQ in Models 1 and 2. Machado et al. demonstrated that PhA is associated not only with physical function but also with QOL in patients with idiopathic pulmonary fibrosis^29^. Azar et al. reported in their study of dialysis patients that PhA is a clinically useful predictive tool because it reflects both physical function and QOL^9^. To the best of our knowledge, this is the first study to investigate the relationship between PhA and QOL in LSS, with results similar to those of previous studies. JOABPEQ is a lumbar spine disease-specific QOL measure and is a highly responsive outcome measure for assessing changes over time in patients with low back pain^21^. EQ-5D, on the other hand, is a widely used comprehensive QOL assessment^30^. The study found that PhA was associated with disease-specific QOL, suggesting that PhA and QOL may be related in patients with LSS. However, a correlation with the EQ-5D, a comprehensive quality of life scale, was found in the bivariate analysis but not in the multiple regression analysis. This may be because the EQ-5D we used was a three-level version, which could provide less information and discrimination than the newly developed five-level version^30^.

Few studies have investigated both QOL measures, and the results of this study seem to confirm the above findings. Moreover, as mentioned earlier, PhA also reflected the physical function (lumbar spine function) of LSS patients in this study. Evaluating both the physical function and QOL of patients and helping patients improve these parameters is the focus of rehabilitation^31^. Therefore, PhA may be a useful tool in physical therapy in patients with LSS.

### Limitations

Some limitations of this study should be considered. First, it was a cross-sectional study, and the causal relationship was unclear because the broader relationship between disease and factors was not known due to the design. In addition, it was a single-center study with a small number of cases with no direct assessment of lumbar function. Moreover, we demonstrated that PhA is associated with QOL and physical function in patients with LSS in this study. However, we did not include healthy subjects or those with lumbar disc herniation. The dysuria or psychological depression seen in LSS patients was not examined. In this study, only preoperative patients were studied; future studies of patients who are unsuitable for surgery and research involving postoperative patients are necessary.

## Conclusion

PhA was associated with physical function and QOL in patients with LSS and may be a useful new tool for preoperative clinical assessment in patients with LSS.

## Data Availability

The datasets generated and/or analysed during the current study are available in the Harvard Dataverse repository, 10.7910/DVN/REBPZ4.
